# A feasibility study: association between gut microbiota enterotype and antibody response to seasonal trivalent influenza vaccine in adults

**DOI:** 10.1002/cti2.1013

**Published:** 2018-03-29

**Authors:** Nick Shortt, Hazel Poyntz, Wayne Young, Angela Jones, Aurélie Gestin, Anna Mooney, Darmiga Thayabaran, Jenny Sparks, Tess Ostapowicz, Audrey Tay, Sally Poppitt, Sarah Elliott, Georgia Wakefield, Amber Parry‐Strong, Jacqui Ralston, Olivier Gasser, Richard Beasley, Mark Weatherall, Irene Braithwaite, Elizabeth Forbes‐Blom

**Affiliations:** ^1^ Medical Research Institute of New Zealand Wellington New Zealand; ^2^ High‐Value Nutrition National Science Challenge Wellington New Zealand; ^3^ Malaghan Institute of Medical Research Wellington New Zealand; ^4^ AgResearch Palmerston North New Zealand; ^5^ Human Nutrition Unit School of Biological Sciences University of Auckland Auckland New Zealand; ^6^ Food Savvy Wellington New Zealand; ^7^ Centre for Endocrine, Diabetes and Obesity Research CCDHB Wellington New Zealand; ^8^ Institute of Environmental Science and Research Limited (ESR) NCBID Upper Hutt New Zealand; ^9^ Wellington School of Medicine University of Otago Wellington New Zealand; ^10^ Present address: Institute of Nutritional Science Nestle Research Centre Lausanne Switzerland

**Keywords:** feasibility study, gut microbiota enterotype, haemagglutinin assay antibody response, seasonal trivalent influenza vaccination

## Abstract

**Objective:**

We investigated the potential feasibility of a randomized controlled trial of a nutritional intervention that may alter human gut microbiota and support immune defence against respiratory tract infection in adults (Proposed Study).

**Methods:**

In total, 125 healthy adults aged 18–64 participated in a 6‐month study that measured antibody response to the seasonal trivalent influenza vaccine. We assessed completion rates, procedure adherence rates and the influence of possible exclusion criteria on potential recruitment into the Proposed Study. We examined whether the gut microbiota could be categorised into enterotypes, and whether there was an association between enterotypes and the antibody response to the influenza vaccine.

**Results:**

The participant completion rate was 97.6% (95% CI 93.1–99.5%). The proportions (95% CI) of participants who may be excluded for antibiotic or corticosteroid use in the 30 days prior to the study, or due to receiving the influenza vaccine in the previous two years were 9.6% (5.1–16.2), 8.0% (3.9–14.2) and 61.6% (52.5–70.2), respectively. All participants were stratified into four gut microbiota enterotypes. There was no association between these enterotypes and the antibody response to the influenza vaccine, although the study was not powered for this outcome.

**Conclusion:**

This study design is suitable for the Proposed Study. The completion rate is likely to be high, although exclusion criteria should be selected with care. Further analyses of gut microbiota composition or function in association with antibody and immune responses are warranted to explore the role of host–microbiota interactions on protective immunity.

## Introduction

Previous investigations have demonstrated a clear relationship between the gut microbiota and the protective antibody response to influenza in animal models,^1^, ^2^ and clinical studies also suggest the human gut microbiota influences the immune response.^3^, ^4^ This raises the possibility that an individual's gut microbiota may affect the strength of their immune response to vaccination.

The major difficulty in dissecting the contribution of the gut microbiota to health and disease is the high variation between and within individuals.^5^ Gut microbiota enterotypes or community types were proposed as a method to classify individuals based on the abundance of certain microbial taxa in fecal samples.^6^ Initially, three enterotypes based on the abundance of *Bacteroides*,* Prevotella* and *Ruminococcus* were described.^6^ More recently, four distinct fecal enterotypes were identified based on complex configurations of numerous microbial taxa, supporting the original findings that the taxa which characterise each enterotype can represent the microbial ecosystem as a whole.^7^ Each of these four enterotypes represented a cluster of relative abundance profiles of five genera including *Bacteroides*,* Prevotella, Alistipes, Fecalibacterium* and Ruminococceae.^7^ Yet whether characterisation of the gut microbiota using this approach can predict disease risk, responsiveness to therapies or responsiveness to vaccines remains to be determined.

Given the mounting evidence of the effect of gut microbial composition on immunity and protective antibody responses, we hypothesised that nutritional concepts supporting appropriate host–microbiota interactions may improve vaccine responsiveness. As a first step, adequately powered observational studies are required to determine whether specific gut microbiota enterotypes are associated with immune responsiveness to vaccination. Randomized controlled trials (RCTs) would then be required to assess this hypothesis, such as a two‐arm parallel‐groups trial in which adults with a gut microbiota enterotype known to be associated with a reduced immune response receive either a placebo, or a nutritional intervention designed to modify gut microbiota composition to a profile that enhances the generation of a protective antibody response. The efficacy of a nutritional concept would be assessed by measuring the response to an immune challenge, in this instance the seasonal trivalent influenza vaccine as compared to placebo control (Proposed Study). A feasibility study is necessary to investigate potential issues that may hamper the success of the Proposed Study.

This feasibility study was designed to inform a number of key aspects of the Proposed Study, including participant completion rate; procedure adherence; the effect of exclusion criteria on recruitment; establishing proportion and stability of each gut microbiota enterotype over time; and if possible, assessing the association between gut microbiota enterotypes and the antibody response (seroconversion and seroprotection) to the Influvac^®^ Vaccine (Mylan, Illinois, USA).

## Results

### Baseline characteristics

**Figure 1:**
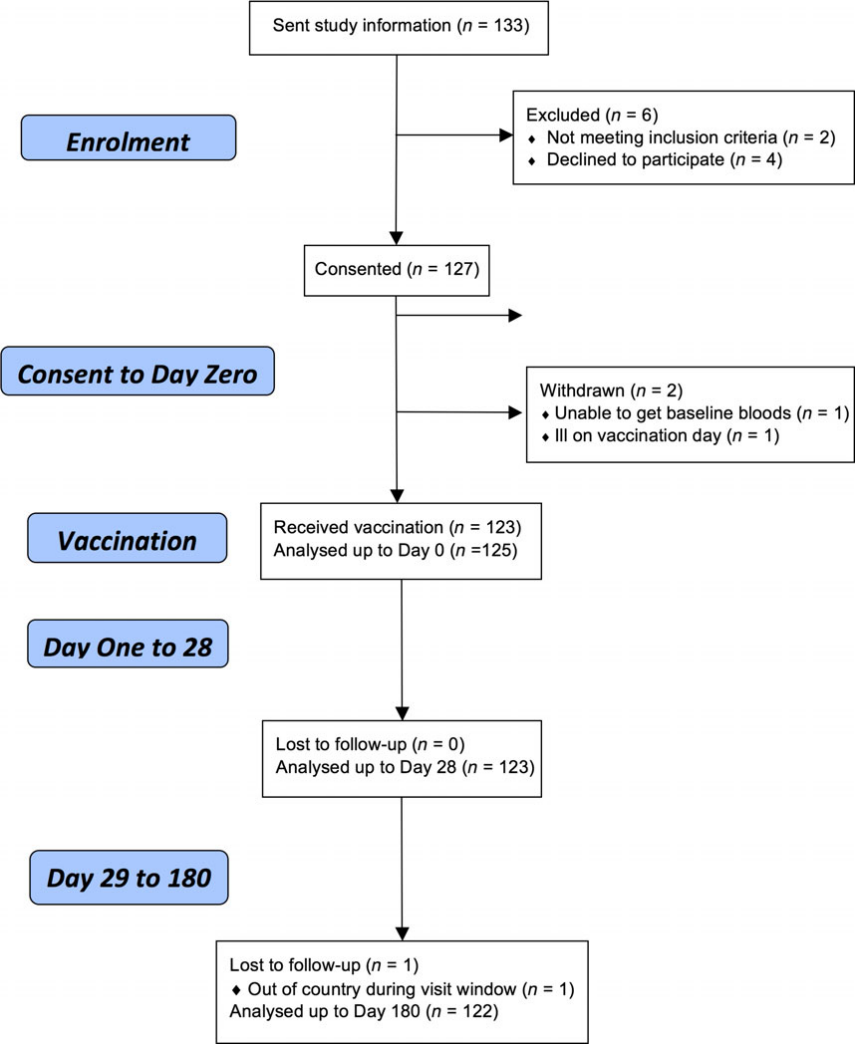
Flow of participants.

The flow of participants is shown in Figure 1, and baseline characteristics are shown in Table 1.

**Table 1:** Participant characteristics

	All (*n* = 125)		Gut microbiota enterotype at day 0 (*n* = 123)							
			A (*n* = 64)		B (*n* = 4)		C (*n* = 23)		D (*n* = 32)	
	Mean (SD)	Range	Mean (SD)	Range	Mean (SD)	Range	Mean (SD)	Range	Mean (SD)	Range
Age	34.5 (12.4)	18–64	33.7 (12.7)	18–64	38.3 (9.6)	28–51	35.1 (11.7)	20–64	35.3 (13.3)	20–64
BMI^a^ (kg/m^2^)	24.8 (4.4)	17.2–41.2	24.2 (4.2)	17.2–38.2	31.7 (8.5)	21.3–41.2	23.5 (3.3)	19.2–31.6	25.9 (3.9)	19.1–38.9
	***N*** **/125 (%)**		***N*** **/64 (%)**		***N*** **/4 (%)**		***N*** **/23 (%)**		***N*** **/32 (%)**	
Proportion female	79 (63.2)		42 (65.6)		2 (50)		15 (65.2)		18 (56.3)	
Ethnicity										
NZ European/European	102 (81.6)		53 (82.8)		2 (50)		20 (87.0)		25 (78.1)	
Maori	7 (5.6)		3 (4.7)		1 (25)		0 (0)		3 (9.4)	
Pacific Islander	1 (0.8)		0 (0)		0 (0)		1 (4.4)		0 (0)	
Asian	7 (5.6)		5 (7.8)		1 (25)		0 (0)		1 (3.1)	
Other	7 (5.6)		3 (4.7)		0 (0)		2 (4.7)		3 (9.4)	
Obesity (BMI^a^ > 30 kg/m^2^)	14 (11.2)		5 (7.4)		2 (50)		1 (4.4)		5 (15.6)	
Caesarean birth	27/122^b^ (22.1)		14/62^b^ (22.6)		2 (50)		5/22^b^ (22.7)		5 (15.6)	
Vegetarian	11/123^b^ (8.9)		8 (12.9)		0 (0)		2 (8.7)		0 (0)	
Diet as an infant										
Primarily breast milk	79/113^b^ (69.9)		44/59^b^ (74.6)		2 (50)		9/17^b^ (52.9)		22/31^b^ (71.0)	
Primarily formula	14/113^b^ (12.4)		3/59^b^ (5.1)		0 (0)		3/17^b^ (17.7)		8/31^b^ (25.8)	
Mix of breast milk and formula	20/113^b^ (17.7)		12/59^b^ (20.3)		2 (50)		5/17^b^ (29.4)		1/31^b^ (3.2)	
Alcohol consumption										
Daily	2/124^b^ (1.6)		2 (3)		0 (0)		0 (0)		0 (0)	
Regularly (3–5 times/week)	40/124^b^ (32.3)		20 (31.3)		0 (0)		6 (26.1)		13 (40.6)	
Occasionally (1–2 times/week)	35/124^b^ (28.2)		20 (31.3)		1 (25)		7 (30.4)		7 (21.9)	
Rarely	40/124^b^ (32.3)		19 (26.7)		3 (75)		9 (39.1)		9 (28.1)	
Never	7/124^b^ (5.6)		3 (4.7)		0 (0)		1 (4.4)		3 (9.4)	
Currently smoking	22 (17.6)		15 (23.4)		1 (25)		2 (8.7)		4 (12.5)	
Diagnosis of IBS	10 (8.1)		9 (14.1)		0 (0)		0/22 (0)		1 (3.1)	
Previous influenza vaccination										
Ever	102 (81.6)		52 (81.3)		4 (100)		20 (87.0)		25 (78.1)	
Last two years	77 (61.6)		42 (65.6)		2 (50.0)		13 (56.5)		19 (59.4)	
CMV positive	58/123^c^ (47.2)		32 (55.2)		2 (50)		11 (47.8)		13 (40.6)	
Antibiotics used between Day −30 and Day 0	12 (9.6)		7 (10.9)		0 (0)		3 (13.0)		2 (6.3)	
Corticosteroids used between Day −30 and Day 0	10 (8.0)		5 (7.8)		0 (0)		3 (13.0)		2 (6.3)	

SD, standard deviation; BMI, body mass index; IBS, irritable bowel syndrome; CMV, cytomegalovirus.

a Self‐reported height and weight were recorded in the Lifestyle Questionnaire and used to calculate BMI.

b Sample size differs from the number provided at the top of the table due to participants selecting ‘not sure’ as an answer or not providing information.

c Change in sample size as data from withdrawn participants not included in this analysis.

### Participant completion

The study was completed by 97.6% (95% CI 93.2–99.5) of the participants, corresponding to a withdrawal rate of 2.4% (Table 2).

**Table 2:** Proportions and 95% confidence intervals for predefined feasibility questions associated with proposed RCT

Feasibility question	*n*/*N* (%)	95% CI
Study completion to Day 28	123/125 (98.4)	93.2–99.5
Study completion to Day 180	122/125 (97.6)	93.1–99.5
Lifestyle Questionnaire completion: Day 0	124/124 (100)	
Food diary completion, 3 days at Day 0	96/123 (78.0)	69.7–85.0
Food diary completion, minimum 5 days at Day 0	53/123 (43.1)	34.2–52.3
Food diary completion, 3 days at Day 28	82/123 (66.7)	57.6–74.9
Food diary completion, minimum 5 days at Day 28	41/123 (33.3)	25.1–42.4
Fecal sample provided: Day 0	124/124 (100)	
Fecal sample inadequately labelled: Day 0	29/124 (23.4)	16.3–31.8
Fecal sample provided: Day 28	123/123 (100)	
Fecal sample inadequately labelled: Day 28	23/123 (18.7)	12.2–26.7
Full blood sampling achieved Day 0	118/124 (95.2)	89.8–98.2
Full blood sampling achieved Day 3	123/123 (100)	
Full blood sampling achieved Day 7	121/122 (99.2)	95.5–100
Full blood sampling achieved Day 28	123/123 (100)	
Full blood sampling achieved Day 180	119/122 (97.5)	92.9–99.5
Blood samples contained sufficient material for analysis Day 0	118/124 (95.2)	89.8–98.2
Blood samples contained sufficient material for analysis Day 3	109/123 (88.6)	81.6–93.6
Blood samples contained sufficient material for analysis Day 7	121/122 (99.2)	95.5–99.8
Blood samples contained sufficient material for analysis Day 28	123/123 (100)	
Blood samples contained sufficient material for analysis Day 180	120/122 (98.4)	94.2–99.8

### Procedure adherence

Adherence to the Lifestyle Questionnaire was 100% (Table 2).

The possible influence of dietary intake beyond the nutritional intervention in the proposed RCT requires collection of these data via participant completion of food diaries. The Day 0 food diary was adequately completed out to three and five days by 78% (95% CI 69.7–85.0) and 43.1% (95% CI 34.2–52.3) of participants, respectively. The Day 28 food diary was adequately completed out to three and five days by 66.7% (95% CI 57.6–74.9) and 33.3% (95% CI 25.1–42.4) participants, respectively. While 100% of the requested fecal samples were provided, 23.4% (95% CI 16.3–31.8) and 18.7% (95% CI 12.2–26.7) of the Day 0 and Day 28 fecal samples, respectively, were inadequately labelled and required the investigator to add participant numbers, dates or times samples were taken. On Day 0 95.2% (95% CI 89.8–98.2) of the blood sample collections were successful. The success rate for blood sample collection was consistently above 95%, and was 100% on Day 3.

### Possible exclusion criteria

The proportion (95% CI) of participants who used antibiotics or corticosteroids in the 30 days prior to Day 0 was 9.6% (5.1–16.2) and 8.0% (3.9–14.2), respectively (Table 3).

**Table 3:** Proportions and 95% confidence intervals for possible exclusion criteria for the proposed RCT

Possible exclusion criteria	*n*/*N* (%)	95% CI
Used systemic antibiotics in the 30 days prior to Day 0	12/125 (9.6)	5.1–16.2
Used systemic antibiotics between Day 0 and Day 180	32/122 (26.2)	18.7–35.0
Used systemic corticosteroids in the 30 days prior to Day 0	10/125 (8.0)	3.9–14.2
Used systemic corticosteroids between Day 0 and Day 180	15/122 (12.3)	7.1–19.5
Influenza vaccine within the last 2 years	77/125 (61.6)	52.5–70.2
Pregnant at Day 0	0/122	
Became pregnant between Day 0 and Day 180	0/122	

Of the 125 participants in the study, 61.6% (95% CI 52.5–70.2) had been vaccinated in the last two years and 18.4% (95% CI 12–26.3%) had never been vaccinated. No pregnant participants were enrolled into the study, and none became pregnant during the study.

### Gut microbiota enterotypes

All participants were able to be mapped to a gut microbiota enterotype on Day 0 and Day 28. The respective proportions are shown in Table 4. 68.3% (59.3–76.4) of participants had the same gut microbiota enterotype at Day 28 as they did at Day 0.

**Table 4:** Proportion of participants with each gut microbiota enterotype at Day 0 and Day 28

		Gut microbiota enterotype Day 28, *n* (%)			
		A 59 (48)	B 6 (4.9)	C 26 (21.1)	D 32 (26.0)
Gut microbiota enterotype, Day 0 *n*/123 (%)	A 64 (52)	51	2	10	1
	B 4 (3.3)	1	2	0	1
	C 23 (18.7)	4	0	10	9
	D 32 (26)	3	2	6	21

### Association between gut microbiota enterotype and antibody response

HAI titres are shown in Table 5. There was no significant association found between Day 0 gut microbiota enterotype and change in GMT, seroconversion or seroprotection rates achieved in response to the vaccine (Table 6).

**Table 5:** Antibody response generated by participants to Influenza vaccination, overall and by gut microbiota enterotype

	All (*n* = 123)		Gut microbiota enterotype at Day 0 (*n* = 123)							
			A (*n* = 64)		B (*n* = 4)		C (*n* = 23)		D (*n* = 32)	
	Mean (SD)	Range	Mean (SD)	Range	Mean (SD)	Range	Mean (SD)	Range	Mean (SD)	Range
Prevaccination HAI titre										
H1N1^a^	116.5 (231.3)	5–1280	134.3 (270.7)	5–1280	41.3 (30.7)	5–80	145.9 (267.6)	5–1280	69.1 (80.4)	5–320
H3N2^b^	114.7 (307.6)	5–2560	164.0 (408.5)	5–2560	92.5 (152.6)	5–320	77.2 (145.4)	5–640	45.9 (70.9)	5–320
B^c^	7.8 (6.4)	5–40	7.6 (5.7)	5–40	6.3 (2.5)	5–10	9.1 (8.1)	5–40	7.5 (6.7)	5–40
Postvaccination HAI titre										
H1N1^a^	252.0 (291.8)	10–1280	249.2 (293.3)	10–1280	82.5 (61.3)	10–160	381.7 (399.1)	20–1280	185.6 (161.7)	10–640
H3N2^b^	188.8 (339.5)	5–2560	201.8 (305.0)	5–1280	211.3 (293.4)	5–640	267.2 (572.6)	5–2560	103.8 (105.6)	10–320
B^c^	26.3 (62.4)	5–640	31.0 (81.7)	5–640	18.8 (15.5)	5–40	27.4 (33.6)	5–160	17.0 (28.4)	5–160
Prevaccination logarithm titres										
H1N1^a^	3.75 (1.43)	1.61–7.15	3.79 (1.51)	1.61–7.15	3.34 (1.20)	1.61–4.38	3.96 (1.52)	1.61–7.15	3.58 (1.25)	1.61–5.77
H3N2^b^	3.35 (1.55)	1.61–7.85	3.62 (1.63)	1.61–7.85	3.17 (1.99)	1.61–5.77	3.15 (1.52)	1.61–6.46	2.95 (1.29)	1.61–5.77
B^c^	1.89 (0.49)	1.61–3.69	1.88 (0.47)	1.61–3.69	1.78 (0.35)	1.61–2.30	2.00 (0.58)	1.61–3.69	1.85 (0.49)	1.61–3.69
Postvaccination logarithm titres										
H1N1^a^	4.91 (1.23)	2.30–7.15	4.84 (1.30)	2.30–7.15	4.04 (1.20)	2.30–5.08	5.44 (1.08)	3.00–7.15	4.77 (1.10)	2.30–6.46
H3N2^b^	4.37 (1.34)	1.61–7.85	4.52 (1.31)	1.61–7.15	4.21 (2.07)	1.61–6.46	4.29 (1.65)	1.61–7.85	4.14 (1.04)	2.30–5.77
B^c^	2.61 (0.96)	1.61–6.46	2.64 (1.04)	1.61–6.46	2.65 (0.89)	1.61–3.69	2.91 (0.84)	1.61–5.08	2.32 (0.85)	1.61–5.08
Ratio of geometric means										
H1N1^a^	8.7 (17.7)	0.5–128	8.2 (18.9)	0.5–128	2.0 (0.01)	2–2	11.0 (17.7)	1–64	8.8 (16.4)	1–64
H3N2^b^	7.3 (17.8)	0.5–128	6.9 (18.2)	0.5–128	3.75 (3.1)	1–8	10.0 (26.2)	0.5–128	6.5 (9.0)	1–32
B^c^	3.8 (7.7)	0.5–64	4.4 (9.1)	0.5–64	3.5 (3.3)	1–8	4.1 (6.5)	1–32	2.6 (5.4)	1–32
Seroconversion^d^ at Day 28	***n*** **/123 (%)**		***n*** **/64 (%)**		***n*** **/4 (%)**		***n*** **/23 (%)**		***n*** **/32 (%)**	
H1N1^a^	43 (35.0)		22 (34.4)		0 (0)		11 (47.8)		10 (31.3)	
H3N2^b^	45 (36.6)		20 (31.3)		2 (50)		10 (43.5)		13 (40.6)	
B^c^	20 (16.3)		11 (17.2)		1 (25)		5 (21.7)		3 (9.4)	
All strains	8 (6.5)		4 (6.3)		0 (0)		2 (8.7)		2 (6.3)	
Seroprotection^e^ at Day 28	***n*** **/123 (%)**		***n*** **/64 (%)**		***n*** **/4 (%)**		***n*** **/23 (%)**		***n*** **/32 (%)**	
H1N1^a^	107 (87.0)		54 (84.4)		3 (75.0)		22 (95.7)		28 (87.5)	
H3N2^b^	98 (79.7)		53 (82.8)		3 (75)		17 (73.9)		25 (78.1)	
B^c^	27 (22.0)		15 (23.4)		1 (25)		6 (26.1)		5 (15.6)	
All strains	19 (15.5)		11 (17.2)		0 (0)		4 (17.4)		4 (12.5)	

SD, standard deviation; HAI, hemagglutination inhibition.

a Influenza A (H1N1) A/California/7/2009, X‐181.

b Influenza A (H3N2) A/New Caledonia/71/2014, X257A.

c Influenza B (B/Brisbane/60/2008, wild type).

d Seroconversion: a ≥fourfold increase in HAI titre if prevaccination HAI titre was ≥1:10; or a postvaccination HAI titre of 1:40 if prevaccination HAI titre was <1:10.

e Seroprotection: a postvaccination HAI titre of ≥1:40.

**Table 6:** Associations between gut microbiota enterotype and outcome variables. Estimates and 95% Confidence Intervals for comparisons between gut microbiota enterotype at Day 0 and change in antibody titre (Panel A), Odds Ratios and 95% Confidence Intervals for associations between gut microbiota enterotype at Day 0 and seroconversion to the influenza vaccine (Panel B) and Odds Ratios and 95% Confidence Intervals for associations between gut microbiota enterotype at Day 28 and seroprotection from the influenza vaccine (Panel C), by each component of the influenza vaccine. Note that the individual comparisons should be interpreted very cautiously if the overall *P* value for each association is not significant

Comparison	Influenza A (H1N1)^a^		Influenza A (H3N2)^b^		Influenza B^c^	
	Univariate	Multivariate	Univariate	Multivariate	Univariate	Multivariate
Panel (A) Gut Microbiota Enterotype and change in antibody titre (logarithm scale): Estimate (95% CI)						
A versus B	0.80 (−0.43 to 2.04)	0.56 (−0.39 to 1.51)	0.31 (−1.06 to 1.68)	0.12 (−0.95 to 1.19)	−0.01 (−0.99 to 0.97)	0.13 (−0.83 to 1.09)
A versus C	−0.60 (−1.18 to −0.02)	−0.46 (−0.89 to −0.03)	0.23 (−0.42 to 0.88)	−0.08 (−0.56 to 0.41)	−0.27 (−0.73 to 0.19)	−0.21 (−0.65 to 0.22)
A versus D	0.06 (−0.45 to 0.58)	−0.03 (−0.42 to 0.35)	0.38 (−0.20 to 0.95)	0.004 (−0.44 to 0.44)	0.31 (−0.10 to 0.72)	0.20 (−0.06 to 0.73)
Overall *P* for Enterotype association	0.08	0.10	0.98	0.60	0.17	0.16
Panel B: Gut Microbiota Enterotype and seroconversion^d^ to influenza vaccine: Odds ratio (95% CI)						
A versus B	NA (zero cell count for B)	NA (zero cell count for B)	0.45 (0.06–3.46)	0.61 (0.05–6.85)	0.62 (0.06–6.59)	1.38 (0.07–26.6)
A versus C	0.57 (0.22–1.50)	0.57 (0.17–1.86)	0.59 (0.22–1.57)	0.60 (0.20–1.80)	0.75 (0.23–2.44)	0.74 (0.20–2.44)
A versus D	1.15 (0.46–2.86)	1.64 (0.53–5.08)	0.66 (0.28–1.60)	0.72 (0.26–1.97)	2.01 (0.52–7.77)	2.70 (0.64–11.4)
Overall *P* for Enterotype association	0.15	0.08	0.62	0.79	0.57	0.42
Panel C: Gut Microbiota Enterotype and seroprotection^e^ from influenza vaccine: Odds ratio (95% CI)						
A versus B	1.80 (0.17–19.1)	1.03 (0.06–17.3)	1.61 (0.15–16.9)	3.81 (0.27–53.8)	0.92 (0.09–9.49)	2.44 (0.13–44.4)
A versus C	0.25 (0.03–2.03)	0.20 (0.02–1.80)	1.70 (0.55–5.29)	1.48 (0.45–4.83)	0.87 (0.29–2.59)	0.97 (0.29–3.23)
A versus D	0.77 (0.22–2.68)	0.65 (0.17–2.43)	1.35 (0.47–3.90)	1.57 (0.51–4.84)	1.65 (0.54–5.05)	2.08 (0.63–6.83)
Overall *P* for Enterotype association	0.81	0.71	0.43	0.40	0.77	0.59

CI, confidence interval.

a Influenza A (H1N1) A/California/7/2009, X‐181.

b Influenza A (H3N2) A/New Caledonia/71/2014, X257A.

c Influenza B (B/Brisbane/60/2008, wild type).

d Seroconversion: a ≥ fourfold increase in HAI titre if prevaccination HAI titre was ≥ 1:10; or a postvaccination HAI titre of 1:40 if prevaccination HAI titre was < 1:10.

e Seroprotection: a postvaccination HAI titre of ≥ 1:40.

## Discussion

This feasibility study demonstrated a high retention rate of participants and successful collection of necessary blood samples. Participant completion of the food diary and labelling of fecal samples require improvement. Implementing exclusion criteria of antibiotic as well as systemic corticosteroid use prior to or during the study need to be carefully considered. Limiting recruitment to those who have not been vaccinated in the last two years or those who have never been vaccinated could seriously impair recruitment into the Proposed RCT. This feasibility study was not adequately powered to determine associations between gut microbiota enterotype and antibody response.

### Withdrawal rate

A withdrawal rate of 20% from this feasibility study was anticipated; however, we had a 98% retention rate. This was partly due to the ability of participants to choose their own visit schedule and appointment time from a large selection, ultimately automated using an electronic browser‐based system (http://www.doodle.com, Doodle, Switzerland), and using text message reminders for appointments. Using the lower bound of the calculated CI results in a worst‐case predicted retention rate of 93%, corresponding to a withdrawal rate in the Proposed RCT of 7%.

### Protocol adherence

Procedure adherence results should also be taken into account when determining the sample size for the Proposed RCT. The Lifestyle Questionnaire completion and provision of fecal samples were both 100% and are thus unlikely to impact the initial sample size calculation in this population. However, many other procedures did not achieve the levels of adherence we had anticipated, and approaches to improve adherence or mitigate the effects of nonadherence should be considered.

Food diary completion is the feasibility issue that requires the most consideration in a large RCT with the best completion rate of 78% for the three‐day diary, and the worst completion rate of 33.3% for the five‐day diary. An electronic food diary or smartphone app may encourage real‐time participant reporting and more accurate collection of data. We can be confident participants will provide the fecal samples at multiple time points, but adequate labelling could be problematic. Even using standard phlebotomy equipment, we can conservatively estimate a blood sampling success rate of 90%, which may be improved by specialised phlebotomists and more specialised equipment.

Simply increasing the enrolment sample size by 10% may allow researchers to account for inadequate blood samples, and an addition of a further 7% may mitigate the loss of data caused by participant withdrawals. However, it would be impractical to increase enrolment numbers to allow for the very large proportion (66.7%) that may not be able to adequately complete a five‐day food diary. Therefore, it may be prudent to reassess the role of the food diary in the Proposed RCT, or significantly change the way in which these data are gathered.

### Proposed exclusion criteria

Antibiotics and corticosteroids are both known to affect gut microbiota composition and immune responses.^8^, ^9^, ^10^ It may be desirable to exclude users of these medications from the Proposed RCT. Up to 26% and 12% of our participants reported antibiotic and/or systemic corticosteroid use pre‐Day 0 or between Day 0 and Day 180, respectively. One option may be to stratify participants by prior antibiotic and corticosteroid use in the randomization process rather than excluding these participants from enrolling.

Previous studies have shown a history of influenza vaccination alters antibody responses to subsequent influenza vaccination.^11^, ^12^, ^13^ In this study, 60% of participants had received an influenza vaccination in the last two years, whereas 18% had never been vaccinated. This is despite the lack of a formal free vaccination programme in New Zealand for healthy adults in the age groups we recruited. It is likely an exclusion criterion of ‘ever‐vaccinated’ or vaccinated in the last two years would be detrimental to recruitment. This raises the possibility of using an alternative vaccine that is not usually widely administered, reducing the likelihood of participants having pre‐existing vaccine responsiveness and eliminating this confounding effect.

### Gut microbiota enterotypes

In possibly the first study of this kind in New Zealand, we found that over half of the participants were enterotype A at baseline. The small proportion of participants in the other enterotype categories poses potential problems for recruitment of these SCT types into the Proposed RCT. The proportions of gut microbiota enterotypes found in this study resemble the proportions observed by Ding and Schloss in their study utilising the HMP Consortium sample.^7^ The stability of the enterotype was less in our study then was previously observed by Ding and Schloss using samples from the Human Microbiome Project conducted in the Unites States of America; we observed that 31.7% of the participants had a different gut microbiota enterotype at Day 28 from Day 0. These data support the concept that the gut microbiota enterotype distribution can vary over time and is potentially modifiable to therapeutic intervention.^14^ A continuous variation model would pose issues for a large RCT as there would be no fixed enterotype for the gut microbiota to change from or to, and the impact of certain compositional changes during the initiation or maintenance of the antibody response has yet to be explored.

### Association between gut microbiota enterotype and antibody response

The study was not adequately powered to detect associations between enterotype and antibody responsiveness. However, the study was neither powered nor designed to stringently test this association. The point estimates we have generated may be used in the development of an appropriately powered study to investigate the potential role of host–microbiota interactions on protective antibody responses to influenza in the adult population. Furthermore, associations between antibody responsiveness and other functional microbial consortia (beyond the described enterotypes) may yet exist.

## Methods

In total, 125 healthy adult participants, aged 18–64 years who gave written informed consent were recruited for this study.

Exclusion criteria were as follows:

- a known severe reaction or allergy to any components of the influenza vaccine
- any contraindications to vaccination per recommendations of vaccine manufacturer
- a history of Guillain–Barre Syndrome within six weeks of receiving a previous influenza vaccine
- an impaired immune system that may confound immune response testing; that is, any condition that may impair a participant's immune response through either the condition itself or through the treatment of the condition
- already having received the 2016 seasonal influenza vaccine

Participants attended six visits over a six‐month period (Table 7). At Visit 1 (Day −14 to −7), all participants undertook informed consent and received study materials. The Lifestyle Questionnaire was based on the American Gut arm of the Human Microbiome Project.^15^ The seven‐day food diary was provided by the Human Nutrition Unit (HNU), University of Auckland, New Zealand. Participants collected their own fecal samples using the OMNIgene GUT collection kit (DNA Genotek, Ontario, Canada) after completing the food diary for a minimum of three days.

**Table 7:** Schedule of procedures

	Visit 1 Day −14 to −7	Visit 2 Day 0	Visit 3 Day 3	Visit 4 Day 7	Visit 5 Day 28	Visit 6 Day 180
Informed consent	X					
Lifestyle Questionnaire		X				
Self‐collected fecal samples		X				
Seven‐day food diary		X				
Vaccination		X				
Review of adverse events		X	X	X	X	X
Blood samples			(Number of tubes required)			
HAI^a^ antibody titres		1			1	1
Gene expression and immune cell profiling		8	2	5		3
Full blood count		1				

a Hemagglutination inhibition assay.

At Visit 2 (Day 0), a blood sample for the measurement of antibody titres was collected first and if it was unable to be obtained, then the participant was withdrawn from the study as their baseline antibody titre would not be available for comparative analysis at Visit 5 (Day 28). After all remaining blood samples were obtained, the participants were vaccinated with the Medsafe approved 2016 Influvac^®^ Vaccine containing: A/California/7/2009 (H1N1)pdm09‐like strain (A/California/7/2009, X‐181) 15 μg haemagglutinin; A/Hong Kong/4801/2014 (H3N2)‐like strain (A/New Caledonia/71/2014, X257A) 15 μg haemagglutinin and B/Brisbane/60/2008‐like strain (B/Brisbane/60/2008, wild type) 15 μg haemagglutinin.

### Lifestyle Questionnaire

The Lifestyle Questionnaire could be completed electronically using Wufoo (SurveyMonkey, California, USA) or on paper. Acceptable protocol adherence to the Lifestyle Questionnaire was considered to be a minimum of 90% of the questions answered.

### Food diaries

Food diaries were sent to the HNU and analysed using FoodWorks 7 (Xyris Software, V8, Australia). Acceptable adherence was considered to be a reported daily calorie intake of >1.2 × basal metabolic rate (BMR), using predicted BMR based on sex, age, height and weight of each participant at consent. We assessed adherence for both a 5‐day period and for a 3‐day period.

### Fecal samples

For the fecal samples, acceptable adherence was considered in two ways; whether a sample was provided at all by the participant on Day 0 and Day 28; and whether each fecal sample had been adequately dated and time‐stamped by participants.

For microbiota profiling, DNA was extracted from fecal samples using the Nucleospin Soil kit (Macherey‐Nagel (Düren, Germany) following the manufacturer's instructions. Briefly, 500 mg fecal sample was suspended in lysis buffer and mechanically disrupted using ceramic beads. Proteins and PCR inhibitors were then pelleted with the ceramic beads and the supernatant adjusted to DNA‐binding conditions before being passed over Nucleospin soil column to bind the DNA. Residual substances were then removed by efficient washing and finally, the DNA was eluted in 100 μl RNAse‐free water. DNA yield was assessed using the Quantus^™^ fluorometer (Promega, Madison, WA, USA) and DNA quality measured with the Nanodrop ND‐1000 spectrophotometer (Thermofisher, Waltham, MA, USA).

Amplification of the V4 region of the 16S rRNA gene followed by 2 × 250 bp sequencing on the MiSeq platform was performed at NZ Genomics Ltd (NZGL) using the standard Illumina method (https://www.illumina.com/content/dam/illumina-support/documents/documentation/chemistry_documentation/16s/16s-metagenomic-library-prep-guide-15044223-b.pdf).

Amplicon sequences were processed using Qiime 1.8. Paired‐end reads were quality filtered using a Q30 cut‐off and chimeric sequences identified using the USEARCH method against the Greengenes alignment (version 13_8) were removed. OTUs were picked at 97% similarity using the UCLUST method, and representative sequences were assigned taxonomies using the RDP classifier.

We then assigned different gut microbiota enterotypes based on taxonomic groups,^7^ using the following criteria:

- Samples with *Bacteroides* proportions in the upper 90% (10–100th percentile) of the samples are assigned enterotype D.
- Then, samples that had *Prevotella* proportions in the upper 60% (40–100th percentile) were assigned to enterotype C. This includes all the samples that were previously classified as enterotype D that meet these criteria; therefore, they were reassigned to enterotype C.
- Then, samples that had *Bacteroides* proportions in the upper 40% (60–100th percentile) were assigned to enterotype A, including samples previously assigned to D and C, which met these criteria.
- Then, samples that had Prevotella proportions in the upper 10% (90–100th percentile) were labelled enterotype D. This includes samples previously assigned to enterotypes D, C and A, which now met these criteria.
- Remaining unclassified samples were assigned to enterotype B.

### Hemagglutination inhibition (HAI) assays

Adequate adherence to blood sampling was considered to be 100% of planned samples obtained for the visit day. Influenza‐specific antibody titres in serum were quantified using the HAI assay. Serum was separated from whole blood samples by centrifugation then stored at −70°C until use. HAI assays were performed for each influenza strain by the National Centre for Biosecurity & Infectious DiseaseUpper Hutt, New Zealand.

Briefly, serum samples were treated with receptor‐destroying enzyme and heat inactivated to remove non‐specific agglutinins. The samples were titrated out twofold, in duplicate, across a microtitre plate from 1:10 to 1:10240; then, four Haemagglutinating Units of the appropriate Antigen was added. After a 30‐min incubation step, 1% Guinea pig red blood cells (RBC) were added to all wells, including serum controls and test controls and allowed to settle for an hour. Plates were read manually and titre endpoints determined as the last well where RBC agglutination was inhibited. Results are accepted if all controls (Serum and Antigen) gave expected results.

### Outcome measures

The primary outcome measure of this feasibility study was the completion rate of participants in the Proposed Study.

Secondary outcome variables were as follows:

- The proportions of each protocol procedure that was adequately completed (procedure adherence).
- The impact of possible exclusion criteria on recruitment into the proposed RCT. Specific considerations included the proportion of participants who took any antibiotics or any corticosteroids within 30 days of Day 0, or between Day 0 and study completion; the proportion of participants who had received an influenza vaccine in the previous 2 years; and the proportion of participants who were pregnant prior to, or became pregnant during the study.
- The proportion of participants who could be successfully mapped to any of the four prespecified gut microbiota enterotypes at Day 0 and Day 28, and those participants who had the same gut microbiota enterotype at Day 28 as at Day 0.
- The association between gut microbiota enterotype on Day 0 and response to the influenza vaccine. Either geometric mean titre (GMT), or by proportions of participants deemed to be seroprotected (postvaccination HAI titre of ≥40^16^) or seroconverted (a ≥fourfold increase in HAI titre from baseline or a postvaccination HAI titre of four times the lower detection limit if the baseline HAI titre is below the lower detection limit^17^).

### Sample size

Recruiting 100 participants would allow calculation of proportions with a 95% confidence interval of ± 10%. We enrolled 125 participants as we anticipated a 20% withdrawal and dropout rate was possible due to the number of visits required and procedures involved.

### Statistics

Proportions were calculated with a 95% confidence interval (CI) and exact binomial confidence intervals for dichotomous variables were calculated using the Clopper–Pearson method.^18^

Logistic regression was used to explore the association between gut microbiota enterotype and postvaccination GMT/seroconversion/seroprotection using anova for univariate analysis. Older age was associated with lower antibody titres for H1 and H3 (*P *<* *0.001 and *P *=* *0.04, respectively). BMI was associated with lower antibody titres of H1, (*P *=* *0.015) and vaccination within the previous two years was associated with lower B titres (*P *<* *0.001), and so ancova for the multivariate analysis included these variables. Normality assumptions for absolute values of HAI titres were not met, so HAI values were logarithm transformed.

SAS version 9.4 (SAS Institute, North Carolina, USA) was used.

The study was approved by the New Zealand Central Health and Disability Ethics Committee, reference number 15/CEN/207, and prospectively registered with the Australia New Zealand Clinical Trials Registry (ACTRN12615001365550).

## Conclusion

The feasibility of this design for use in a large RCT was supported by the results obtained in this study. There was a low participant withdrawal rate, and with the exception of the food diaries, adherence to procedures was acceptable. Choice of exclusion criteria relating to influenza vaccination history should be carefully considered as it is likely to increase screening numbers substantially. In addition, stratification by use of systemic corticosteroids, antibiotics and vaccination history may be preferable. Further observational studies should be undertaken to fully explore the potential of the gut microbiota to augment antibody responsiveness and improve vaccine efficacy, as a basis for RCTs of dietary interventions.

## Author contributions

- Study concept and design: N Shortt, H Poyntz, I Braithwaite and E Forbes‐Blom
- Acquisition of Data: N Shortt, H Poyntz, W Young, A Jones, A Gestin, A Mooney, D Thayabaran, J Sparks, T Ostapowich, A Tay, S Poppitt, S Elliott, G Wakefield, A Parry‐Strong, J Ralston, I Braithwaite and E Forbes‐Blom
- Drafting of the manuscript: N Shortt, I Braithwaite, E Forbes‐Blom, R Beasley and O Gasser
- Critical revision of the manuscript for important intellectual content: all authors
- Statistical analysis: M Weatherall

## Ethics approval and consent to participate

The study was approved by the New Zealand Central Health and Disability Ethics Committee, reference number 15/CEN/207, and prospectively registered with the Australia New Zealand Clinical Trials Registry (ACTRN12615001365550). All participants gave informed consent prior to undertaking any study procedures.

## Guarantor

Dr I Braithwaite and Mr N Shortt had access to all the data on the study and take responsibility for the integrity of the data and accuracy of the data analysis.

## Transparency declaration

The lead authors affirm that the manuscript is an honest, accurate and transparent account of the data being reported; that no important aspects of the analysis have been omitted; and that any discrepancies from the analysis as planned (and, if relevant, registered) have been explained.

## Data sharing statement

Data sets used and/or analysed are available from the corresponding on request. Consent was not obtained from participants for data sharing, but the presented data are anonymised and the risk of identification is low.

## Conflict of interest

The authors declare no conflict of interest.
